# Associations between defense mechanisms and life satisfaction among North Korean refugees

**DOI:** 10.1186/s12991-021-00339-1

**Published:** 2021-03-09

**Authors:** Chang Woo Lee, Jooyoung Lee, Jin Yong Jun, So Hee Lee, So Young Yu, Juhyun Park, Seog Ju Kim

**Affiliations:** 1 Department of Psychiatry, Sungkyunkwan University School of Medicine, Samsung Medical Center, 81 Ilwon-ro, Kangnam-gu, Seoul, 06351 Korea; 2 Department of Research Planning, Mental Health Research Institute, National Center for Mental Health, Seoul, Republic of Korea; 3 Department of Psychiatry, National Medical Center, Seoul, Republic of Korea; 4 Department of Psychology, University At Buffalo, Buffalo, NY USA

**Keywords:** Refugees, Defense mechanisms, Life satisfaction

## Abstract

**Background:**

The present study investigated associations between defense mechanisms and life satisfaction among North Korean refugees living in South Korea.

**Methods:**

A total of 178 North Korean refugees completed the Korean version of the Defense Style Questionnaire, a revised version of the Ways of Thinking of North Korean Defectors scale, the Center for Epidemiologic Studies-Depression Scale, and the State–Trait Anxiety Inventory. Multiple stepwise regression analysis was performed to investigate the defense mechanisms associated with North Korean refugees’ life satisfaction in South Korea.

**Results:**

Among defense mechanisms, denial most strongly predicted higher overall and economic satisfaction among North Korean refugees living in South Korea (*β* = 0.145, *p* < 0.01; *β* = 0.137, *p* = 0.03, respectively) after controlling for age, gender, anxiety, depression, and number of traumatic events experienced. Furthermore, resignation predicted lower overall (*β* = −0.206, *p* < 0.001) and economic satisfaction (*β* = −0.134, *p* = 0.02). However, the association between resignation and life satisfaction was not significant after controlling for depression, anxiety, and number of traumatic events experienced.

**Conclusions:**

Specific defense mechanisms such as high denial and low resignation were associated with life satisfaction in South Korea among North Korean refugees. Our findings suggest that refugees’ psychological defense mechanisms may affect their satisfactory resettlement.

## Background

According to the United Nations High Commissioner for Refugees, there were 20.4 million refugees worldwide in 2018 [1]. Most of these people have experienced major transitions in systems of government, cultural environments, and socioeconomic status, including employment [2]. Furthermore, refugees are commonly exposed to unexpected traumatic events such as imprisonment, torture, and exile. This experience of multiple traumatic events makes refugees more vulnerable to psychological distress and psychiatric disorders [3–6], which may impede their successful adaptation and stable resettlement [7, 8].

Defense mechanisms are defined as an individual’s responses to emotional conflicts and external stresses [9]. They are unconscious psychological process protecting individual’s mind by preventing conscious awareness of internal or external threatening. Defense mechanisms are known to play important roles for successful psychological adaptation. The response to particular situations depends on which defense mechanism is invoked. Individuals using adaptive defense mechanisms respond appropriately to the overall context of a situation. Responding with maladaptive defense mechanisms, however, could worsen stressful situations and aggravate negative emotions and perceptions [10, 11].

Defense mechanisms may also affect psychological adaptation in those who experience serious traumatic events, such as refugees. Interrelationship between defense mechanism, psychological adaptation, and traumatic experiences has been reported. Memory of childhood abuse and immature defense style were reported to be associated with adulthood psychopathology [12]. In addition, immature defense styles were reported to mediate the path between posttraumatic stress disorder and psychological distress among university students [13]. Defense mechanisms can affect people’s assessment of themselves, such as their self-esteem and sense of self-efficacy [14, 15]. Defense mechanisms may also affect psychiatric symptoms such as depression [16, 17]. Some defense mechanisms may be adaptive in terms of appropriately dealing with stressful conditions [18]. However, others may threaten self-esteem and exacerbate psychiatric symptoms [19, 20], which could affect life satisfaction [21, 22].

Previous studies of life satisfaction among refugees focused on differences in the conditions encountered by refugees and characteristics of new settlements. In the case of Bosnian refugees in Norway, for example, the positive reactions of existing residents to refugees affected refugees’ life satisfaction [23]. Another study showed that the closer refugees’ ethnicity was to that of existing residents, the higher was immigrants’ life satisfaction [24]. However, how refugees psychologically accept a new environment during resettlement has rarely been studied. To the best of our knowledge, no study has examined the relationship between refugees’ predominant defense mechanisms and specific domains of life satisfaction.

Most North Korean refugees left North Korea to escape economic difficulties and because of their dissatisfaction with the regime. By 2018, more than 32,000 North Korean refugees had settled in South Korea, most of whom entered South Korea after 2000 [25]. North Korean refugees have typically suffered traumatic situations such as imprisonment, torture, sexual assault, separation from their families, forced repatriation, and arrests by authorities, as well as having witnessed public executions and shootings [26–28]. After settlement in South Korea, they have experienced prejudice, an unfamiliar culture, and rapid socioeconomic changes [29]. This series of painful experiences can undermine life satisfaction among North Korean refugees living in South Korea [30–32].

Based on previous studies of North Korean refugees and other refugee populations, we hypothesized that the specific defense mechanisms employed by North Korean refugees would be correlated with their life satisfaction in South Korea. In this study, the primary objective was to investigate which specific defense mechanisms were predominantly associated with specific life satisfaction domains among North Korean refugees. We also aimed to identify psychiatric and demographic variables that might affect the relationship between defense mechanism and life satisfaction.

## Methods

### Participants

The initial participants were 213 North Korean refugees living in South Korea who were recruited through advertisements. Data from 35 subjects who did not complete questionnaires were excluded from the analyses. Finally, 178 North Korean refugees were included in this study (43 males, 135 females). The average age was 37.84 ± 11.40 years. Age and gender were not significantly different between the excluded and included subjects. The mean time since their defection from North Korea was 2.99 ± 3.79 years at the time of study participation. The mean time they had lived in countries other than South and North Korea was 2.75 ± 3.64 years. The mean time of settlement in South Korea was 2.81 ± 2.59 years. This study was approved by the Institutional Review Board at Myeongji Hospital, Kwandong University. All participants were provided with a complete description of the study, and written informed consent was obtained from them all.

To check for previous traumatic events, the Trauma Exposure Check List for North Korean Refugees, which is based on the Traumatic Experiences Scale for North Korean Defectors, was used [17, 30]. This self-report questionnaire evaluates previously experienced traumatic events that have frequently occurred among North Korean Refugees. It covers 13 types of traumatic events experienced in North Korea and 16 types experienced during defection. Trauma events in the questionnaire included torture, severe battery, threats to life, famine/cold/accidents, rape, human trafficking, arrest/imprisonment, and witnessing public executions.

### Assessments

#### Defense style questionnaire

The Korean version of the Defense Style Questionnaire (K-DSQ) was used to identify the participants’ defense mechanisms. The K-DSQ is a self-report questionnaire based on the Defense Style Questionnaire (DSQ) [18, 33]. The DSQ includes 78 questions about 25 defense mechanisms. Some defense mechanisms represented by fewer than three questions on the DSQ were excluded from the K-DSQ because of reliability considerations. The K-DSQ consists of 66 questions measuring 16 defense mechanisms: acting out, consumption, denial, fantasizing, resignation, inhibition, reaction formation, passive aggression, projection, splitting, sublimation, undoing, withdrawal, omnipotence, humor, and isolation.

#### Life satisfaction in South Korea

To assess life satisfaction in South Korea, we used a revised version of the Ways of Thinking of North Korean Defectors scale [34]. Life satisfaction in South Korea was considered an indicator of successful adaptation to an unfamiliar society and culture. This self-report measure consists of five domains: economic, occupational, medical, educational, and overall satisfaction. The overall domain covers areas such as cultural or interpersonal experiences not addressed by the other four domains. Each item is rated on a 5-point Likert scale. A higher score indicates greater satisfaction in that domain.

#### Depression and anxiety

The Korean version of the Center for Epidemiologic Studies-Depression Scale (CES-D), which consists of 20 items covering depression symptoms, was used to assess depression. The State–Trait Anxiety Inventory (STAI) was used to assess anxiety. The STAI has two components, with 20 questions evaluating state anxiety (STAI-S) and 20 questions evaluating trait anxiety (STAI-T). In the current study, we only used state anxiety scores, as trait anxiety may be less affected by external environmental factors such as defection or settlement.

### Statistics

To explore group differences in quantitative variables, t tests were used. To investigate the relationship between life satisfaction and defense mechanisms, multiple stepwise regression analysis was conducted. To identify defense mechanisms more determinant on the prediction of life satisfaction, stepwise selection method was used for minimizing effects of co-linearity among 16 defense style. Three different regression models were used with modification of covariates. Model 1 was the multiple regression model setting 16 defense mechanisms as independent variables and five satisfaction levels as dependent variables. Model 2 added age and gender as independent variables to those in Model 1. Model 3 also added CES-D, STAI-S, and number of traumatic events experienced as independent variables to those of Model 2. Independent variables that were significant in the previous models were considered as independent variables in the next model. All statistical analyses were performed using SPSS ver. 21 (IBM Corp., Armonk, NY, USA). For all analyses, statistical significance was defined as two-tailed p values < 0.05.

## Results

### Demographic and clinical characteristics

Table 1 shows the demographic characteristics of participants and clinical information including defense mechanisms, life satisfaction, and CES-D and STAI-S scores. We found no significant association between the defense mechanisms studied and age. CES-D scores increased with age (*r* = 0.16, *p* = 0.03). Males were more satisfied than females with education in South Korea (*t* = 2.78, *p* < 0.01). Significant gender differences were seen in defense mechanisms of consumption, inhibition, sublimation, and withdrawal. Males used consumption as a defense mechanism more than females did (*t* = -4.04, *p* < 0.001), whereas females used inhibition (*t* = 2.18, *p* = 0.03), sublimation (*t* = 2.39, *p* = 0.02), and withdrawal (*t* = 1.99, *p* = 0.048) as defense mechanisms more than males. North Korean refugees who participated in the study experienced an average of 7.04 ± 5.48 traumatic events.

**Table 1:** Demographic and clinical characteristics of North Korean Refugees (*n* = 178)

	Mean ± SD
Demographic characteristics	
Age, years	37.84 ± 11.40 (range: 19–73)
Female, *n* (%)	135 (76%)
Life satisfaction in South Korea	
Economic	3.05 ± 1.10
Occupational	2.94 ± 1.17
Medical	3.93 ± 0.94
Educational	3.90 ± 0.94
Overall	3.51 ± 1.03
Defense Style Questionnaire	
Acting out	3.33 ± 1.39
Consumption	2.74 ± 1.30
Denial	2.70 ± 1.24
Fantasizing	2.70 ± 1.29
Resignation	3.12 ± 1.43
Inhibition	3.57 ± 1.11
Reaction formation	3.26 ± 1.24
Passive aggression	2.89 ± 1.08
Projection	2.50 ± 0.92
Splitting	3.74 ± 1.16
Sublimation	3.74 ± 1.24
Undoing	3.51 ± 1.24
Withdrawal	3.80 ± 1.67
Omnipotence	3.79 ± 1.47
Humor	3.92 ± 1.42
Isolation	2.58 ± 1.15
CES-D	19.83 ± 12.14
STAI-S	44.45 ± 11.75
Number of traumatic events experienced	7.04 ± 5.48

*CES-D* the Center for Epidemiological Studies-Depression scale, *STAI-S* the State-Trait Anxiety Inventory-State

### Life satisfaction and defense mechanisms

In simple correlation, overall satisfaction was significantly correlated with denial (r = 0.227, *p* < 0.01), resignation (*r* = − 0.219, *p* < 0.01), withdrawal (*r* = − 0.160, *p* < 0.05), and humor (*r* = 0.239, *p* < 0.01). Economical satisfaction was significantly correlated with denial (*r* = 0.195, *p* < 0.01), sublimation (*r* = 0.181, *p* < 0.05), and humor (*r* = 0.183, *p* < 0.05). Resignation was correlated with economical satisfaction only in non-significant level (*r* = − 0.124, *p* = 0.098). Educational satisfaction was significantly correlated with reaction formation (*r* = 0.148, *p* < 0.05). Consumption was correlated with economical satisfaction only in non-significant level (*r* = − 0.134, *p* = 0.076). None of the defense mechanisms were significantly correlated with medical or occupational satisfaction (Table 2).

**Table 2:** Correlation efficient between life satisfaction and defense mechanism in North Korean Refugees

	Economic satisfaction	Occupational satisfaction	Medical satisfaction	Educational satisfaction	Overall satisfaction
Acting out	− 0.060	0.065	− 0.024	− 0.110	− 0.045
Consumption	− 0.090	− 0.078	− 0.100	− 0.134	− 0.144
Denial	0.195**	0.112	0.104	0.112	0.227**
Fantasizing	− 0.021	0.025	0.052	− 0.061	− 0.090
Resignation	− 0.124	− 0.060	− 0.039	− 0.113	− 0.219**
Inhibition	0.040	0.096	0.121	0.073	0.040
Reaction formation	0.141	0.102	0.141	0.148*	0.143
Passive aggression	0.013	0.132	0.067	− 0.042	− 0.105
Projection	− 0.098	− 0.105	− 0.055	− 0.112	− 0.134
Splitting	0.018	0.061	0.003	− 0.014	0.015
Sublimation	0.181*	0.129	0.110	0.135	0.107
Undoing	− 0.042	0.070	0.062	− 0.024	− 0.077
Withdrawal	− 0.127	− 0.062	0.013	− 0.068	− 0.160*
Omnipotence	− 0.021	0.069	− 0.023	0.041	− 0.002
Humor	0.183*	0.139	− 0.015	0.064	0.239**
Isolation	− 0.120	0.066	0.053	− 0.088	− 0.132

*p < 0.05, **p < 0.01,

In Model 1 of multiple stepwise regression analysis, greater use of denial (*β* = 0.189, *p* < 0.01) and lower use of resignation (*β* = −0.181, *p* < 0.01) significantly predicted higher overall satisfaction. In Model 2, after controlling for age and gender, denial and resignation were still significantly associated with overall satisfaction (*β* = 0.241, *p* < 0.001; *β* = − 0.206, *p* < 0.001, respectively). In Model 3, after additionally controlling for depression, anxiety, and trauma, only denial significantly predicted overall satisfaction (*β* = 0.145, *p* < 0.01) (Table 3).

**Table 3:** Associations between life satisfaction and defense mechanisms in North Korean Refugees in stepwise multiple regression model

Life satisfaction	Defense mechanism	Model 1	Model 2	Model 3
Overall	Denial	0.189**	0.241***	0.145**
	Resignation	− 0.181**	− 0.206***	*ns*
Economic	Denial	0.203**	0.203**	0.137*
	Resignation	− 0.131*	− 0.134*	*ns*
Educational	Reaction formation	0.142*	*ns*	*ns*
	Consumption	− 0.127*	*ns*	*ns*

Model 1: dependent variable: life satisfaction; independent variables: defense mechanisms

Model 2: dependent variable: life satisfaction; independent variables: defense mechanisms, age, and gender

Model 3: dependent variable: life satisfaction; independent variables: defense mechanisms, age, gender, depression, anxiety, and number of traumatic events experienced

*β* is provided only if *p* value was < 0.05

Excluded variables in stepwise model were not shown or marked as *ns*

*ns* non-significant

^*^p < 0.05, **p < 0.01, ***p < 0.001

With regard to economic satisfaction, in Model 1, greater use of denial predicted higher (*β* = 0.203, *p* < 0.01) and greater use of resignation predicted lower economic satisfaction (*β* = −0.134, *p* = 0.02). In Model 2, after controlling for age and gender, denial and resignation were significant predictors of economic satisfaction, as in Model 1 (*β* = 0.203, *p* < 0.001; *β* = −0.134, *p* = 0.02, respectively). In Model 3, after additionally controlling for depression, anxiety, and trauma, denial was the only significant predictor of economic satisfaction (*β* = 0.137, *p* = 0.03).

In Model 1, reaction formation showed a positive association (*β* = 0.142, *p* = 0.01), and consumption showed a negative association (*β* = − 0.127, *p* = 0.02) with educational satisfaction. Reaction formation and consumption were no longer significant in Model 2 after controlling for age and gender. None of the defense mechanisms significantly predicted medical or occupational satisfaction.

## Discussion

This study investigated associations between specific defense mechanisms and life satisfaction among North Korean refugees in South Korea. The results showed that greater use of denial predicted higher overall and economic life satisfaction among North Korean refugees in South Korea, even after controlling for depression, anxiety, and number of traumatic events experienced. Low resignation also predicted higher overall and economic life satisfaction, although this association was affected by depression and anxiety.

As suggested by our primary hypothesis, specific defense mechanisms affected specific domains of life satisfaction. Denial affected life satisfaction independently of psychiatric symptoms such as depression and anxiety. The positive relationship between denial and life satisfaction may indicate that denial leads individuals to rate their subjective life satisfaction as high [35, 36]. Denial is often described as a defense mechanism that protects one’s subjective perception of traumatizing and stressful aspects of given situations [37, 38]. Because of individuals who use denial refuse to recognize the reality of painful conditions, denial could be regarded as a maladaptive defense mechanism. However, some have suggested that denial is not always maladaptive. Rather, it may serve to temporarily distance the individual from stressful events, allowing the person to feel safe and satisfied. One study reported that denial was grouped with other adaptive defense mechanisms including humor, omnipotence, and sublimation in a factor analysis of the DSQ [33]. Especially, in unusually tragic situations, several studies have shown that denial served as an adaptive coping strategy [37, 38]. However, self-reported denial may not be associated with satisfaction in the unconsciousness. Unaccompanied refugee minors were reported to have both higher distress and higher denial of distress [39]. In another study on preadolescent victims of a lightning strike, the association between higher denial and lower clinical upset was found in self-reports, while this association was not observed by parents’ report [40]. These findings may suggest that life satisfaction associated with denial may not be consistent with the deep-seated unconscious satisfaction.

Resignation entails passively accepting a situation by simply not coping with negative events that feel uncontrollable. In a previous study of patients diagnosed with lung cancer, those who showed resignation in the course of accepting the disease reported low quality of life and a negative mood [41]. The present study showed that North Korean refugees who used resignation to cope with stressors had lower overall and economic life satisfaction. However, the effects of resignation on life satisfaction seemed to be influenced by depression or anxiety. Giving up on dealing with situations and holding negative expectations for the future may cause a negative mood that then leads to low life satisfaction.

Among the domains of life satisfaction considered in these North Korea refugees, the overall and economic domains were most strongly related to defense mechanisms; educational, medical, and occupational satisfaction showed less association with defense mechanisms. Generally, the acceptability of education and medical care in a new society depends on the social systems of that community rather than on one’s personal characteristics. In South Korea, the government offers basic educational and medical opportunities equally to all people, including North Korean refugees, so it makes sense that satisfaction with education and medical care was less affected by refugees’ defense mechanisms. On the other hand, as economic status can differ among individuals in a capitalist society such as South Korea, satisfaction with one’s economic status is more strongly associated with individual characteristics. The average period spent in South Korea was less than 3 years. During this period, most refugees participated in a training course or educational program at a government agency before they officially took a job. For this reason, occupational satisfaction would not be likely to be associated with individuals’ defense mechanisms.

If refugees express high life satisfaction even under painful circumstances, it is important to keep in mind that they may be using denial as a defense mechanism. For refugees who use denial and claim a high level of life satisfaction, determining which aspects of denial are helpful to them may provide some insight for mental health practitioners working with refugees. When a refugee expresses dissatisfaction with the situation, it would be helpful to check for depression and anxiety related to feelings of resignation. For refugees who frequently use resignation as a defense, highlighting its negative aspects, such as avoidance of reality, may help to improve the patient's psychiatric symptoms and life satisfaction.

The current study has several limitations. First, this study was conducted using a cross-sectional design. Hence, sufficient evidence for causal relationships between defense mechanisms and life satisfaction is lacking. Furthermore, we did not obtain information about psychiatric symptoms such as depression and anxiety before the traumatic events considered here. Therefore, the effects of such symptoms on life satisfaction in South Korea could not be investigated in this study. In addition, as the associations between defense styles and life satisfaction may be changed during settlement period of refugees, future study to explore their associations after many years of settlement would be helpful. Second, there may be potential biases in recruiting North Korean refugees, which may be induced by differences in the willingness to participate study or the accessibility to study information. Over 80% of North Korean refugees in South Korea are female, so it is not surprising that the current study had more female than male participants. Thus, this study may not have sufficiently considered the experiences of male North Korean refugees. Third, even though life satisfaction is subjective, it can be affected by objective economic variables such as employment and income. Refugees’ objective economic status might indicate how well they have adapted to the new social system. In the present study, interpretation of the relationships between objective status, defense mechanisms, and life satisfaction is limited.

## Conclusions

This study examined relationships of defense mechanisms with life satisfaction among North Korean refugees living in South Korea. Specific defense mechanisms, such as more frequent use of denial and less frequent use of resignation, were associated with overall and economic life satisfaction. Denial affected life satisfaction independently of depression, anxiety, and traumatic experiences. However, the association between resignation and life satisfaction seemed to be mediated by depression or anxiety. The present findings suggest that understanding the type of defense mechanisms employed may help in understanding experiences of life satisfaction and potentially facilitate successful resettlement of refugees.
